# Perinatal risk factors of renal outcome in former extremely low birth weight neonates

**DOI:** 10.1007/s00431-024-05730-0

**Published:** 2024-08-24

**Authors:** Marieke Colleman, Jan A. Staessen, Karel Allegaert, Anke Raaijmakers

**Affiliations:** 1 Department of Development and Regeneration, University of Leuven, Louvain, Belgium; 2 Research Association Alliance for the Promotion of Preventive Medicine, Mechelen, Belgium; 3 Biomedical Research Group, Faculty of Medicine, University of Leuven, Louvain, Belgium; 4 Department of Pharmaceutical and Pharmacological Sciences, University of Leuven, Louvain, Belgium; 5 Department of Hospital Pharmacy, Erasmus Medical Center, Rotterdam, The Netherlands; 6 Department of Paediatric Nephrology, Sydney Children’s Hospital Randwick, Sydney Children’s Hospital Network, Randwick, NSW Australia; 7 School of Women’s and Children’s Health, University of New South Wales, Randwick Clinical Campus, Randwick, NSW Australia

**Keywords:** Extremely low birth weight, ELBW, Estimated glomerular filtration rate, eGFR, Cystatin C

## Abstract

Former Extremely Low Birthweight (ELBW) neonates suffer from adverse renal and cardiovascular outcomes later in life. Less is known about additional perinatal risk factors for these adverse outcomes which we have investigated in this study. We compared renal outcome between ELBW children and controls, to find perinatal risk factors for poorer renal outcome and to unveil associations between kidney function and blood pressure. This study included 93 former ELBW children and 87 healthy controls with a mean age of 11 years at assessment. We measured cystatin C-based estimated glomerular filtration rate (eGFR) and blood pressure. Blood pressure and eGFR levels were compared between cases and controls. We subsequently investigated perinatal risk factors for adverse outcome amongst ELBW children. ELBW children have significantly higher blood pressure (mean SBP percentile 75^th^ *vs.* 47^th^, *p* <0.001) and lower mean eGFR (94 *vs.* 107 ml/min/1.73 m^2^, *p* = 0.005) compared to the control group. Elevated blood pressure did not correlate with perinatal characteristics and none of them had microalbuminuria. ELBW children with eGFR <90 ml/min/1.73 m^2^ were ventilated longer (17 vs. 9 days, *p* = 0.006), more frequently male (OR = 3.33, *p* = 0.055) and tended to suffer more from intraventricular hemorrhage (40% vs. 15.8%, *p* = 0.056). There was no association between blood pressure and kidney dysfunction. *Conclusions*: Understanding risk profiles for unfavorable outcomes may help to identify children at increased risk for kidney dysfunction. Poorer eGFR was associated with longer ventilation, male sex, and intra-ventricular hemorrhage but not with blood pressure. This knowledge can lead to safer neonatal therapeutic regimens for ELBW infants, a more intensive follow-up and earlier treatment initiation for children at highest risk.

**Table:** 

**What is Known:** • *Extremely Low Birthweight (ELBW) neonates suffer later in life from adverse renal and cardiovascular outcomes.* • *Perinatal risk factors that further predict the individual risk for adverse outcomes are not well known.*
**What is New:** • *Poorer eGFR in adolescence was associated with male sex, longer ventilation and intra-ventricular hemorrhage at birth but not with blood pressure.* • *Former ELBW infants had higher blood pressures compared to controls, but no microalbuminuria.* • *This knowledge can lead to potential precision medicine, safer neonatal therapeutic regimens for ELBW infants, a more intensive follow-up and earlier treatment initiation for children at highest risk.*

**Supplementary Information:**

The online version contains supplementary material available at 10.1007/s00431-024-05730-0.

## Introduction

Extremely low birthweight (ELBW, i.e. birthweight below 1000 g) children are at increased risk of developing adverse cardiovascular and renal outcomes in later life [1–3]. Their improved survival necessitates more focused research on long-term outcome aspects [3, 4].

In cardiovascular dysfunction, renin–angiotensin–aldosterone system (RAAS) alterations in the vascular tree could play a role in the development of hypertension [2, 5, 6]. RAAS is modified during preterm birth, possibly because of the inability of the heart and kidneys to respond properly to the postnatal transition [7, 8]. Preterm birth alters vascular development, leading to functional and structural changes possibly involving endothelial function, arterial narrowing, and pulse wave propagation. Moreover, in vitro endothelial progenitor cells of preterm infants show an inability to form qualitative capillary networks [5].

ELBW children show smaller kidney length and a lower (estimated) glomerular filtration rate (eGFR) [6, 9]. Nephrogenesis arises through branching morphogenesis, similarly to the vascular tree, lungs, retina, and pancreas [10, 11]. Not only does nephrogenesis continue until 34–36 weeks of gestation, over 80% of nephrons are formed during the final trimester of pregnancy. Organs formed by branching morphogenesis may be particularly vulnerable to the detrimental results of preterm birth. Accelerated maturation of the kidneys following preterm birth likely leads to more morphologically abnormal glomeruli and glomerular enlargement in preterm kidneys [12]. According to the Developmental Origins of Health and Disease concept, kidney insults in early life may lead to altered organ function and morphology and eventually lead to chronic kidney disease (CKD) [2, 13].

ELBW children differ from healthy controls in cardiovascular outcome, leading to increased morbidity and mortality in later life [2, 4]. There is, however, a poorly understood variety in outcome amongst these children. We hypothesize that perinatal determinants could influence future renal health. In this study, we analyzed data of 93 ELBW children and 87 healthy controls and compared their renal outcome at a mean age of 11 years. We aimed to identify perinatal risk factors amongst ELBW infants that help elucidate future kidney function decline. As a proof of concept, we developed a model to explore future kidney function in ELBW children using perinatal determinants.

## Methods

This research is part of the PREMATCH studies [14] and was conducted in accordance with the Helsinki Declaration for investigation in human subjects. The study protocol was approved by the Ethics Committee of the University Hospital of Leuven, Belgium, and may be accessed via ClinicalTrials.gov; number NCT02147457.

The 93 cases were recruited amongst a previously described cohort of 140 ELBW survivors born between 2000 and 2005 in the University Hospital of Leuven [15]. All children were invited to participate in the follow-up study. Of all 140 invited children, 93 could be contacted and consented to participate (66.4%). The 87 controls were matched for age and gender and were either a friend (*n* = 41) or recruited from an elementary school near the research center in Eksel, Belgium (*n* = 46). They were not selected for birth weight but had to be > 37 weeks of gestation. All participants were invited to a series of examinations at the study’s research center [14].

Blood pressure was calculated as the average of three consecutive measurements on a single home visit using the auscultatory method on a standard mercury sphygmomanometer. Blood pressures were all adjusted for age and height and converted to percentiles. Elevated systolic/diastolic blood pressure (SBP/DBP) was defined as average SBP/DBP ≥ 90th percentile; systolic/diastolic hypertension was defined as average SBP/DBP ≥ 95th percentile. Elevated blood pressure was defined as the occurrence of either elevated SBP or DBP. Hypertension was defined as the occurrence of either systolic or diastolic hypertension, as per the American Association of Pediatrics’ guidelines [16].

Kidney function was assessed by cystatin C-based eGFR. Serum cystatin C was determined with a particle-enhanced turbidimetric immunoassay (Tina-quant cysC Generation 2 assay on a COBAS Integra 400 system from Roche Diagnostics). Subsequent eGFR was calculated following conversion of cystatin C values using the Caucasian-Asian-Pediatric-Adult formula [17]:

$$\mathrm{eGFR}=130\bullet\mathrm{Cystatin\;C}^{-1.069}\bullet\mathrm{age}^{-0.117}-7$$

Kidney function decline was defined as an eGFR below 90 ml/min/1.73 m^2^. Body surface area (BSA) was calculated by the Mosteller Formula [18]:

$$\mathrm{BSA}=\sqrt{\frac{\mathrm{height}\;\left(\mathrm{cm}\right)\bullet\mathrm{weight}\;(\mathrm{kg})}{3600}}$$

Microalbuminuria as a marker for glomerular dysfunction was defined as a urinary albumin excretion of > 30 mg in a 24 h urine collection.

### Statistical analysis

Statistical analysis was performed with MatLab (MathWorks, version R2020a). Any outliers >3SD were omitted. Antenatal and postnatal characteristics were described by median and range if continuous, and by mean incidence if dichotomous. Normality of data was assessed. With regards to all outcomes, ELBW children were first compared with controls using a *t*-test or Mann–Whitney *U* test as appropriate. Perinatal characteristics of ELBW children with kidney function decline or elevated blood pressures were subsequently compared to ELBW children with normal outcome. In the case of continuous variables, Mann–Whitney *U* testing was used whereas Fisher’s exact test was used for dichotomous variables. Spearman’s ρ was used to evaluate the association between risk factors and continuous perinatal factors, whereas point-biserial ρ was used for dichotomous perinatal factors. Results were considered significant with a two-sided *p*-value of <0.05; trends were defined as a *p*-value between 0.05 and 0.1.

## Results

The cohort consisted of 93 ELBW children and 87 controls. Baseline characteristics can be found in Table 1. Investigated perinatal characteristics of the study population can be found in Table S1. Blood samples were successfully taken from 59 out of 93 cases and 71 out of 87 controls (failure to collect blood was due to refusal, too low sample volume or collection failure after 1 or 2 attempts). Urinalysis for microalbuminuria was available for 82 cases.

**Table 1:** Characteristics of the study population at age 11

Characteristics at age 11	ELBW (*n* = 93)	Controls (*n* = 87)	*p*-value
Age (years) (mean, 95% CI)	11.6 (9.5–14.7)	11.1 (9.3–14.9)	0.0141
Length (cm) (mean, 95% CI)	145 (124–168)	147 (129–182)	0.1684
Weight (kg) (mean, 95% CI)	33.4 (21.4–67.8)	37.5 (25.7–71.4)	0.0041
Body surface area (m^2^) (mean, 95% CI)	1.17 (0.85–1.77)	1.23 (0.97–1.83)	0.0004

*ELBW*, extremely low birth weight; *CI*, confidence interval

**Fig. 1:**
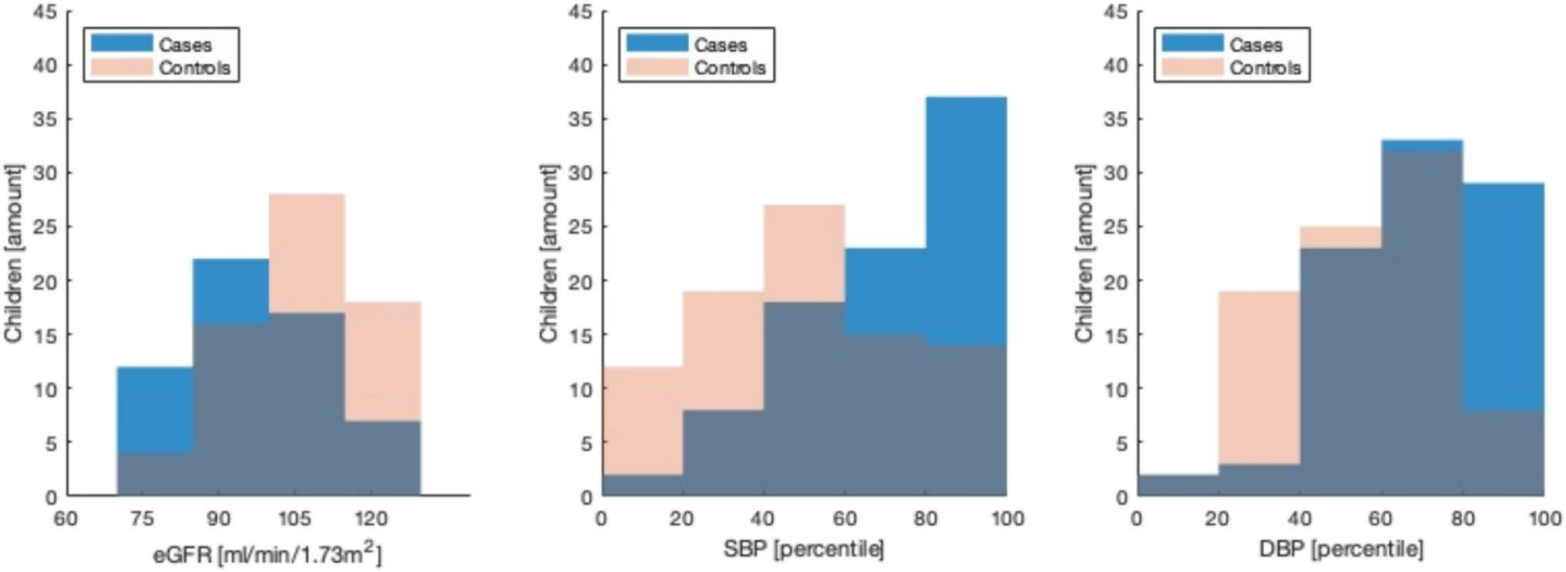
Estimated glomerular filtration rate (eGFR), systolic blood pressure (SBP) and diastolic blood pressure (DBP) distributions for ELBW children and controls. The purple area represents the overlap between cases and controls

Blood pressures were significantly higher amongst ELBW children than controls (Fig. 1, Table 2). Average SBPs were in the 75^th^ percentile for cases compared to the 47^th^ percentile for controls (*p* <0.001). Average DBPs were in the 68th percentile for cases versus the 54th percentile for controls (*p* <0.001). Elevated SBP and systolic hypertension occurred significantly more frequently amongst ELBW children than controls (OR = 6.15, *p* <0.001 and OR = 13.6, *p* = 0.002). Elevated DBP was far less prevalent than elevated SBP and found to not significantly differ between ELBW children and controls. While 38.6% of ELBW children had elevated blood pressures (either elevated SBP or DBP), this was only 10% for the controls (OR = 5.5, *p* <0.001).

**Table 2:** Outcomes of ELBW children and controls at the age of 11 years

	ELBW	Controls	*p*-value
**Kidney function**	***n* = 59**	***n* = 71**	
Mean eGFR [ml/min/1.73 m^2^] (SD)	94.1 (13.3)	106.5 (15.1)	** <0.001**
eGFR < 90 ml/min/1.73 m^2^ (%)	34.5	12.7	**0.005**
**Blood pressure**	***n* = 93**	***n* = 87**	
SBP (percentile)	75 (23)	47 (18)	** <0.001**
DBP (percentile)	68 (25)	54 (19)	** <0.001**
Elevated SBP (%)	27.3	5.6	** <0.001**
Hypertensive SBP (%)	13.6	1.2	**0.002**
Elevated DBP (%)	7.8	3.5	0.33
Hypertensive DBP (%)	1.1	0	-
Elevated blood pressure (%)	38.6	10.3	** <0.001**
Hypertension (%)	13.6	1.2	**0.002**

*ELBW*, extremely low birth weight; *eGFR*, estimated glomerular filtration rate; *SBP*, systolic blood pressure; *DBP*, diastolic blood pressure

On average, ELBW children had an eGFR of 94.1 ml/min/1.73 m^2^, while healthy controls had an eGFR of 106.5 ml/min/1.73 m^2^ (*p* < 0.001, Fig. 1). Amongst ELBW children, 34.5% had reached a kidney function decline (defined as eGFR < 90 ml/min/1.73 m^2^) and 12.7% in the control group (Table 1).

### Perinatal risk factors in relation to outcomes in adolescence

None of the investigated perinatal factors showed an association with elevated SBP. Former ELBW children with an elevated DBP were born at a higher gestational age (29.5 vs. 27.2 weeks, *p* = 0.001). These children were subsequently discharged at a significantly lower weight (1786 vs. 2240 g, *p* = 0.01). Sex, being small for gestational age (SGA), birthweight, intra-ventricular hemorrhage (IVH), broncho-pulmonary dysplasia (BPD), retinopathy of prematurity (ROP), postnatal ibuprofen use, or steroids did not associate with elevated SBP or DBP. No direct correlation was observed between SBP or DBP percentiles and eGFR for the population of ELBW children.

Male ELBW children had a tendency of developing eGFR < 90 ml/min/1.73 m^2^ in comparison to female ELBW children (OR = 3.33, p = 0.055). ELBW children who developed an eGFR < 90 ml/min/1.73 m^2^ received significantly longer ventilation therapy (17 vs. 9 days, *p* = 0.006). They additionally tended to suffer more frequently from IVH (40% vs. 15.8%, *p* = 0.056) (Supplementary Table S2). Birthweight, being SGA and gestational age played no role. Ibuprofen and steroids had no effect on the outcome of ELBW children (Supplementary Table S2). None of the ELBW children had developed microalbuminuria by age 11 (*n* = 82, data not shown).

The most significant perinatal determinants for an unfavorable renal outcome in ELBW children were used to create a model that could illuminate their risk of a lower eGFR by age 11, defined in the study as a single measurement of eGFR <90 ml/min/1.73 m^2^. A point was assigned for sex (male), ventilation therapy (> 10 days) and intraventricular hemorrhage IVH (any), representing vulnerability through sex, exposure to intensive treatment and the occurrence of neonatal comorbidities.

**Fig. 2:**
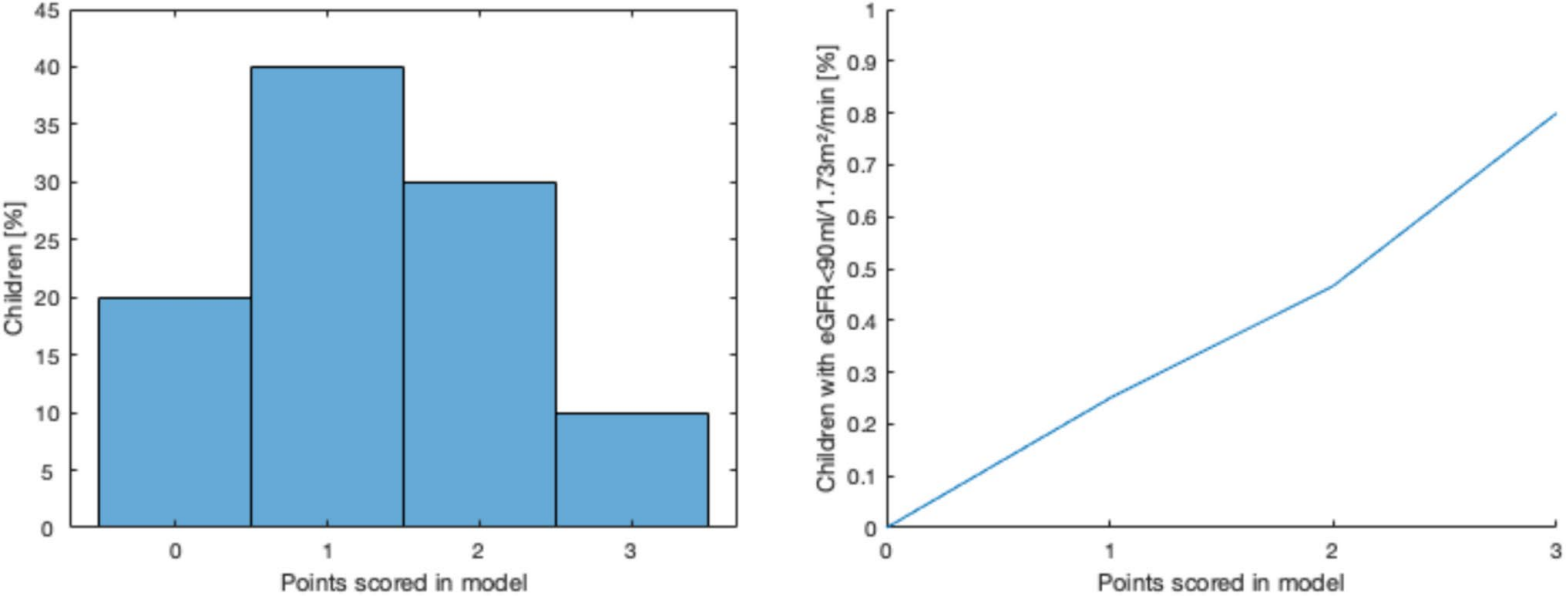
Modelling renal outcome in former ELBW children using three perinatal determinants: male sex, mechanical ventilation therapy > 10 days and occurrence of any intraventricular hemorrhage. Presence of these determinants would give children 0, 1, 2 or 3 points. The percentages on the y-axis refer to the number of children within these groups having a certain amout of points (left panel) or a reduced kidney function (right panel). Perinatal determinants were chosen as these characteristics correlated most significantly with kidney function decline (defined as eGFR < 90 ml/min/1.73 m^2^)

As a proof of concept, we modelled the risk for kidney function decline (defined as eGFR < 90 ml/min/1.73 m^2^), using the criteria sex (male), ventilation (> 10 days) and IVH (any). Amongst ELBW children 34.5% progressed to kidney function decline. This risk progresses linearly between 0 and 80% varying on the points scored in the model (Fig. 2).

## Discussion

This study investigated kidney health of former ELBW neonates and searched for perinatal factors associated to adverse outcome. Overall, ELBW children have a poorer kidney function compared to controls. We also showed in a model that perinatal risk factors were associated with these outcomes. Most perinatal determinants correlated only weakly with blood pressure and kidney function. Renal health is the result of complex interactions between premature physiology and perinatal determinants, potentially influenced by mechanisms including developmental programming and an impaired branching morphogenesis. Despite this complexity, some associations between perinatal determinants and future health could be withheld.

We confirmed that ELBW children are at an increased risk of developing hypertension [2, 19, 20]. ELBW children with elevated SBP had similar perinatal determinants as children with normal SBP. We conclude that perinatal determinants do not seem to modulate the risk to develop elevated blood pressures in ELBW children at age 11. Accelerated weight gain during the first months and years of life has also been associated with elevated blood pressure [21]. However, during their stay at the neonatal ward ELBW children with elevated blood pressures had not gained weight faster than the other ELBW children in our cohort. Hypertension in childhood is frequently considered a kidney problem [7]. However, we found no correlation between blood pressure and kidney function in ELBW children at the age of 11 years. Our results could implicate that elevated blood pressure has at least partly a different cause than kidney function decline.

The main risk factor associated with a poorer renal outcome amongst ELBW children was duration of ventilation therapy. Historical data have shown that mechanical ventilation increases intrathoracic pressure in the acute phase and therefore could suppress urinary output affecting kidney function [22]. Animal research has indeed shown ventilation to cause impaired postnatal glomerular growth [23]. We hypothesize that ventilation therapy could function as second hit in ELBW children and could reflect respiratory disease severity. Intraventricular hemorrhage showed a trend towards being a risk factor for poorer renal outcome amongst ELBW children. Possibly, an association of IVH to poorer renal outcome could arise from the common development of glomeruli and the (intracranial) vascular network through branching morphogenesis [10]. Interestingly, the effects of being small for gestational age, birthweight and gestational age on kidney function were limited in ELBW children. Neither ibuprofen nor perinatal steroids (both pre- as well as postnatal) influenced long-term renal outcome, an important consideration for their safety assessment in premature infants [9, 24]. It should be noted that none of the ELBW children had developed microalbuminuria by the age of 11. This might indicate that proteinuria only appears as a later risk factor in kidney function decline. However, a similar cohort has shown microalbuminuria in 12% of cases in adolescence [25], but the origin of that cohort was mixed (population selected on gestational age not birth weight and Caucasian-African American; current study birth weight driven and Caucasian only).

We developed an explorative model similar to Schmidt and colleagues [26] to investigate renal outcome in former ELBW children at age 11 by using the three strongest risk factors found during analysis: male sex, the presence of IVH and a prolonged ventilation therapy (> 10 days). The risk of developing kidney function decline increased linearly with the amount of perinatal risk factors. Neonatal comorbidities such as ROP, BPD and IVH have already shown to predict neurological outcome in former ELBW children [27]. This model is intended as a proof of concept that factors such as the perinatal environment and complicating neonatal comorbidities can elucidate long-term outcomes too. However, these finding should be validated through further studies.

A limitation of the study is its low sample size, due to which existing associations were less likely to reach significance. Despite careful interpretation, due to multiple testing some associations could be coincidental findings, so that results should be read as exploratory. Moreover, selection bias could have occurred as analyzed (*n* = 93) and not-analyzed (*n* = 47) participants differed on several antenatal parameters as previously discussed [28, 29]. However, since differences are in both directions (for example less ventilation days but more pre–eclampsia in the analyzed children), we assume that selection was at random. Additionally, the kidney reduced function could not be defined as CKD, as it requires a decreased kidney function for at least three months. A small portion of the controls (12.7%) also had a reduced kidney function. This group was not further investigated, but we assume this is due to a normal variation in this group as displayed in Fig. 1 (Bell curves for both cases as well as controls). Also, a final diagnosis of hypertension could not be confirmed either as it requires measurements on three separate visits. In creating a model for exploring long-term renal outcome with simple perinatal determinants, we have set a proof of concept that could serve as an exemplary to model other non-neurodevelopmental outcomes. The model includes mechanical ventilation and IVH, and it is possible that these risk factors are linked. Future work could focus on merging multiple datasets to find more evidence for perinatal risk factors regarding adverse renal outcome in former ELBW children.

In conclusion, risk profile assessment for unfavorable renal outcome may help to identify those children at increased risk. This knowledge can lead to safer neonatal therapeutic regimens for ELBW infants (for example avoidance of nephrotoxins and early treatment initiation of AKI), more intensive follow-up and earlier screening initiation for children at highest risk (i.e. blood pressure checks, proteinuria assessment). Moreover, risk profiles may help uncover the pathophysiological processes causing these poorer outcomes and these could be different for different organ systems. More epidemiological research is needed to guide early screening programs and potential precision medicine.

## Supplementary Information

Below is the link to the electronic supplementary material.

Supplementary file1 (DOCX 29 KB)

## Data Availability

No datasets were generated or analysed during the current study.

## Abbreviations

CKD: Chronic kidney disease
DBP: Diastolic blood pressure
eGFR: Estimated glomerular filtration rate
ELBW: Extremely low birth weight
IVH: Intra-ventricular hemorrhage
ROP: Retinopathy of prematurity
SBP: Systolic blood pressure
SGA: Small for gestational age
