# Supplementary material for: Perinatal risk factors of renal outcome in former extremely low birth weight neonates
Source: Eur J Pediatr. 2024 Aug 24;183(11):4685–91. doi: 10.1007/s00431-024-05730-0 (PMC11473622; doi:10.1007/s00431-024-05730-0)
Supplement: Supplementary file 1 — Supplementary file1 (DOCX 29 KB) [file 431_2024_5730_MOESM1_ESM.docx]

**Supplementary Tables**

Table **S1**: **Perinatal characteristics of the extremely low birth weight (ELBW) population**.

| Perinatal characteristics | ELBW (n=93) |
| --- | --- |
| Sex (female : male ratio) | 1 : 1.04 |
| Small for gestational age (%) | 39% |
| Gestational age (weeks) (mean and range) | 27 (23 to 33) |
| Birth weight (g) (mean and range) | 815 (430 to 1000) |
| Postnatal steroids (% received) | 51% |
| Ibuprofen (% received) | 49% |
| Oxygen therapy (days) (mean and range) | 36.5 (1 to 164) |
| Ventilation therapy (days) (mean and range) | 7 (0 to 131) |
| Time until full enteral (days) (mean and range) | 33.5 (12 to 143) |
| Hospitalization duration (days) (mean and range) | 82 (13 to 183) |
| Discharge weight (g) (mean and range) | 2348 (860-3225) |
| IVH (% present) | 23% |
| BPD (% present) | 15% |
| ROP (% present) | 19% |

Bronchopulmonary dysplasia (BPD) was defined as either oxygen need for at least 28 days, oxygen therapy past 36 weeks postmenstrual age, or ventilation therapy at 36 weeks postmenstrual age. Intraventricular hemorrhage (IVH) was defined as any intraventricular hemorrhage. Retinopathy of prematurity (ROP) was defined as ROP ≥ stage 3.

Table **S2**: **Risk factor analysis of ELBW children for blood pressure and kidney function.**

|  | Blood pressure | | | | | | Kidney function | | |  |
| --- | --- | --- | --- | --- | --- | --- | --- | --- | --- | --- |
|  | **eSBP**  **(n=23)** | **nSBP**  **(n=64)** | **p** | **eDBP**  **(n=7)** | **nDBP**  **(n=80)** | **p** | **eGFR <90**  **(n=7)** | **eGFR >90**  **(n=52)** | **p** | |
| Sex (girls) | 41.6 | 50.0 | 0.63 | 42.8 | 49.4 | 1 | 25 | 52.3 | 0.055 | |
| SGA (%) (mean) | 50.0 | 38.1 | 0.34 | 71.4 | 37.8 | 0.11 | 35 | 31.5 | 0.32 | |
| Gestational age (weeks) (mean) | 27.8 | 27.4 | 0.40 | 29.5 | 27.2 | **0.001** | 27.3 | 27.2 | 0.89 | |
| Birthweight (grams) (mean) | 797 | 794 | 0.93 | 838 | 791 | 0.38 | 800 | 792 | 0.82 | |
| IVH (% present) (mean) | 16.7 | 25.4 | 0.57 | 14.2 | 24.4 | 1 | 40 | 15.8 | 0.056 | |
| BPD (% present) (mean) | 20.8 | 12.5 | 0.33 | 0 | 15.7 | 0.58 | 15 | 15.8 | 1 | |
| ROP (% present) (mean) | 16.7 | 17.7 | 1 | 0 | 19.8 | 0.34 | 25 | 10.8 | 0.25 | |
| Ibuprofen (% received) | 43.4 | 50 | 0.63 | 33.3 | 50.6 | 0.68 | 65 | 57.9 | 0.78 | |
| Steroids (% received) | 43.4 | 52.4 | 0.63 | 14.2 | 51.9 | 0.11 | 60 | 47.4 | 0.42 | |
| Oxygen (days) (mean) | 39 | 37 | 0.78 | 18 | 40 | 0.09 | 47 | 36 | 0.24 | |
| Ventilation (days) (mean) | 13 | 10 | 0.42 | 7 | 12 | 0.38 | 17 | 9 | **0.006** | |
| Time to full enteral feeds (days) (mean) | 41 | 38 | 0.62 | 33 | 40 | 0.40 | 42 | 35 | 0.22 | |
| Hospitalization (days) (mean) | 84 | 79 | 0.55 | 66 | 83 | 0.15 | 89 | 77 | 0.058 | |
| Discharge weight (grams) (mean) | 2227 | 2172 | 0.62 | 1786 | 2240 | **0.01** | 2307 | 2175 | 0.27 | |

Estimated glomerular filtration rate (eGFR) <90 ml/min/1.73m^2^; eSBP or nSBP: elevated or normal systolic blood pressure; eDBP or nDBP: elevated or normal diastolic blood pressure; ELBW, extremely low birth weight; SGA, small for gestational age. Bronchopulmonary dysplasia (BPD) was defined as either oxygen need for at least 28 days, oxygen therapy past 36 weeks postmenstrual age, or ventilation therapy at 36 weeks postmenstrual age. Intraventricular hemorrhage (IVH) was defined as any IVH. Retinopathy of prematurity (ROP) was defined as ROP ≥ stage 3.
